# Biological Models of Oxidative Purine DNA Damage in Neurodegenerative Disorders

**DOI:** 10.3390/antiox14050578

**Published:** 2025-05-11

**Authors:** Chryssostomos Chatgilialoglu

**Affiliations:** 1 Center for Advanced Technologies, Adam Mickiewicz University, 61614 Poznań, Poland; chachr@amu.edu.pl or chrys@isof.cnr.it; 2 Istituto per la Sintesi Organica e la Fotoreattività, Consiglio Nazionale delle Ricerche, 40129 Bologna, Italy

**Keywords:** DNA damage, oxidative stress, reactive oxygen species (ROS), free radicals, hydroxyl radical, neurodegeneration, 5′,8-cyclopurine, 8-oxo-purine, isotope dilution LC–MS/MS, DNA repair

## Abstract

Most DNA damage caused by oxidative metabolism consists of single lesions that can accumulate in tissues. This review focuses on two classes of lesions: the two 8-oxopurine (8-oxo-Pu) lesions that are repaired by the base excision repair (BER) enzyme and the four 5′,8-cyclopurine (cPu) lesions that are repaired exclusively by the nucleotide excision repair (NER) enzyme. The aim is to correlate the simultaneous quantification of these two classes of lesions in the context of neurological disorders. The first half is a summary of reactive oxygen species (ROS) with particular attention to the pathways of hydroxyl radical (HO^•^) formation, followed by a summary of protocols for the quantification of six lesions and the biomimetic chemistry of the HO^•^ radical with double-stranded oligonucleotides (ds-ODN) and calf thymus DNA (ct-DNA). The second half addresses two neurodegenerative diseases: xeroderma pigmentosum (XP) and Cockayne syndrome (CS). The quantitative data on the six lesions obtained from genomic and/or mitochondrial DNA extracts across several XP and CS cell lines are discussed. Oxidative stress contributes to oxidative DNA damage by resulting in the accumulation of cPu and 8-oxo-Pu in DNA. The formation of cPu is the postulated culprit inducing neurological symptoms associated with XP and CS.

## 1. Introduction

DNA is vulnerable to oxidative insults originating either through the metabolism of oxygen or through environmental sources (e.g., ionizing radiation, UV light, and chemical mutagens), causing DNA strand breaks and chemical modifications of nucleobases [1,2]. Most DNA damage induced through oxidative metabolism consists of single lesions, although multiple lesions, such as tandem or clustered lesions and DNA/DNA or DNA/protein crosslinking, are also reported. Thousands of these lesions accumulate within the mammalian cell genome each day [3]. The damage is particularly deleterious because they cause heritable mutations to daughter cells. Enzymatic systems are known to remove the majority of DNA lesions and maintain the integrity of the genome [3,4]. However, enzymatic deficiencies can give rise to the accumulation of damage to cellular components that are linked to specific pathologies, including neurodegeneration [5]. For a comprehensive overview of oxidatively induced DNA damage, including quantification methods (in vitro and in vivo), repair mechanisms, and biological consequences, please refer to the two-volume set of books recently published [6].

In this review, two groups of purine DNA damage by reactive oxygen species (ROS) are compared, the 8-oxopurine (8-oxo-Pu) and 5′,8-cyclopurine (cPu) lesions, which are repaired by the base excision repair (BER) and nucleotide excision repair (NER) enzymes, respectively. cPu, as transcriptional blocking lesions, have been identified as molecular defects in neurodegenerative processes [7]. The first part of the review highlights the endogenous formation of HO^•^ radicals within the ROS system, the mechanistic aspects for forming these lesions, and their interconnection in biomimetic models. In the second part, emphasis is given to the experimental protocols utilized for quantification of these purine lesions, the role of BER and NER enzymes in repairing these lesions, and the comparison of the literature data of these lesions in some biological models of neurodegenerative disorders.

Molecular oxygen (O_2_) is not just a benign participant in cellular metabolism and function; it also has a dark side when ROS production becomes potentially harmful. Hypoxia, a condition of reduced oxygen supply, has been observed during the aging process and the onset of neurodegeneration. Hypoxia is associated with the pathogenesis of numerous neurological disorders, including Alzheimer’s, Parkinson’s, and other age-related neurodegenerative diseases. The ROS involvement and the mechanisms by which this occurs are not well understood [8,9]. This review also examines the role of O_2_ in biomimetic and biological models, highlighting the need for further research in this area.

## 2. Reactive Oxygen Species (ROS) and Pathways of Hydroxyl Radical Formation

ROS are produced during physiological intracellular metabolism, and their roles in biology are related to redox signaling [10,11,12,13,14]. Various cellular compartments produce ROS, including mitochondria, peroxisomes, cytosol, and the endoplasmic reticulum [15]. ROS encompass various radicals such as superoxide radical anion (O_2_^•−^), nitric oxide (NO^•^), nitrogen dioxide radical (NO_2_^•^), hydroxyl radical (HO^•^), and carbonate radical anion (CO_3_^•−^), which operate as one-electron oxidants, as well as small molecules, including hydrogen peroxide (H_2_O_2_), peroxynitrite (ONOO^−^), and hypohalous acids (HOCl or HOBr), which function as two-electron oxidants [16,17,18,19]. At least 90 different genes encode cellular enzymes to handle ROS efficiently. When the ROS system malfunctions, it can contribute to disease. A recent review outlines how ROS may often act as second messengers within a tightly controlled system, rather than solely being toxic precursors of cellular dysfunction, death, and destruction [15].

The event of oxidative stress (OS) is connected with the balance between oxidants and antioxidants; oxidative eustress and oxidative distress are defined under physiological and supra-physiological deviations, respectively [20,21,22,23]. Particularly in oxidative distress, the ROS excess damages various cell components such as proteins, lipids, and DNA, which are implicated in cancer, cardiovascular disease, neurodegenerative disorders, and aging [24]. For example, ROS-induced lipid peroxidation provokes apoptosis, autophagy, ferroptosis [25], two-electron oxidation of methionine moieties in proteins [26], and ~70,000 DNA lesions (chemical modification of DNA) per cell per day in humans [27].

**Figure 1:**
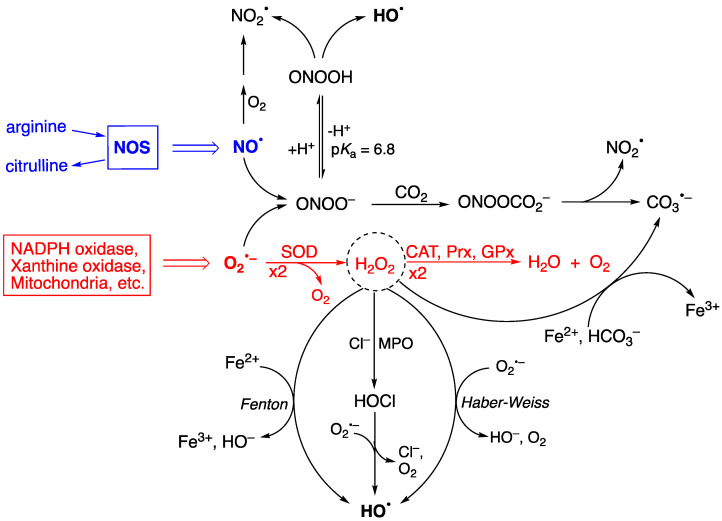
A summary of chemical transformation of the reactive oxygen species (ROS) network. O_2_^•−^ is ubiquitous and continuously formed during normal cellular metabolism by a variety of systems, and NO^•^ is produced by a variety of nitric-oxide synthases (NOS). The superoxide dismutase (SOD) enzymes participate in controlling the production of O_2_^•−^, and the catalase (CAT), glutathione peroxidase (GPx), and peroxiredoxin (Prx) enzymes in their turn are involved in the detoxification of H_2_O_2_ (see reactions in red color). H_2_O_2_ is at the crossroads of several pathways for the formation of HO^•^, the main ones being the Fenton and Haber–Weiss reactions, as well as HOCl formation, mediated by myeloperoxidase (MPO), followed by one-electron oxidation. The protonated form of ONOO^−^ (p*K*_a_ 6.8) spontaneously decomposes, generating HO^•^, whereas the ONOO^−^ acts as a nucleophile with CO_2_ affording an adduct that instantaneously decomposes to NO_2_^•^ and CO_3_^•−^. H_2_O_2_ is also a precursor of CO_3_^•−^ through the reaction with HCO_3_^−^ in the presence of reduced iron (Fe^2+^). Adapted from Ref. [28].

Figure 1 shows the two progenitors of the ROS network [28], which are O_2_^•−^ and NO^•^, and how they are chemically connected. Their concentrations are ~0.1 nM O_2_^•−^ and ~10 nM NO^•^ under physiological conditions but can increase up to 100-fold during inflammatory response, thus affecting severely the integrity of biomolecular heritage. The superoxide dismutase (SOD) enzymes participate in controlling the level of O_2_^•−^, causing its dismutation to H_2_O_2_ and O_2_, in order to maintain the steady-state concentration at picomolar levels [29,30]. H_2_O_2_ is the most abundant ROS in eukaryotes; the intracellular physiological concentration of H_2_O_2_ under oxidative eustress is in the range of 1–10 nM, whereas under oxidative distress, it is larger than 100 nM [31,32]. Catalase (CAT), glutathione peroxidase (GPx), and peroxiredoxin (Prx) participate in the detoxification of H_2_O_2_ by transforming it into water and oxygen, depending upon the site of generation and the specific enzymatic equipment (see Figure 1—red color) [33]. H_2_O_2_ serves a fundamental regulatory function in metabolism, beyond its role as a damage signal [34,35]. At any given moment, the local concentration of H_2_O_2_ in a specific compartment reflects the balance of production and removal dynamics [34].

Figure 1 also shows the importance of H_2_O_2_ in generating radicals. Myeloperoxidase (MPO) uses H_2_O_2_ and anions like Cl^−^ or Br^−^ to generate hypochlorous acid (HOCl) or hypobromous acid (HOBr), respectively. The half-life of HOCl in cells is a few seconds, and its biological properties are different from H_2_O_2_ [11]. Several pathways are involved in the production of HO^•^, the main ones being the Fenton reaction (Fe^2+^ and H_2_O_2_), the Haber–Weiss reaction (O_2_^•−^ and H_2_O_2_), and the reduction of hypohalous acids (HOCl or HOBr) by O_2_^•−^ [28,36,37]. Other biologically critical free radicals can be produced due to ONOO^−^ formation from the diffusion-controlled reaction of O_2_^•−^ and NO^•^ radicals generated during normal cellular metabolism [38,39,40,41,42]. Spontaneous decomposition of the protonated form (ONOOH, p*K*a of 6.8) produces HO^•^ and NO_2_^•^ with a rate constant of 4.5 s^−1^ at 37 °C [38,41,43,44]. The HO^•^ radical is the most reactive of the common ROS and has close to diffusion-limit rates with numerous biomolecules (e.g., with glutathione, *k* = 2.3 × 10^10^ M^−1^ s^−1^) [45]. Its diffusion distance is around 1−5 nm, i.e., less than the width of a 100 kDa protein [11]. It must be considered that the HO^•^ radical can be formed from more stable ROS molecules, like H_2_O_2_, HOCl, and ONOO^−^, having a larger diffusion distance. Therefore, the damaging potential of HO^•^ can be expanded by the long-distance reactivity of its progenitors, becoming more than previously thought.

The ubiquity of CO_2_ and its hydrated form bicarbonate (HCO_3_^−^) in biological systems is well documented, e.g., 25 mM HCO_3_^−^ is in equilibrium with 1.3 mM CO_2_ at pH 7.4. The reaction of ONOO^−^ with CO_2_ affords the adduct ONOOCO_2_^−^ with a relatively high rate constant (k = 5.8 × 10^4^ M^−1^s^−1^), followed by a rapid decomposition (<100 ns) to NO_2_^•^ and CO_3_^•−^ [38,43,44]. The reduction potential of CO_3_^•−^/CO_3_^2−^ is 1.59 V, which makes CO_3_^•−^ a milder single-electron oxidant. It is also reported that the Fenton reaction, in the presence of HCO_3_^−^, also produces CO_3_^•−^ as the predominant species rather than the HO^•^ radical [46,47,48]. Given the uncertain nature of the cellular labile iron pool [49], the contribution of Fenton chemistry to ROS generation is likely to be context-dependent [11].

A complex and well-developed antioxidant system protects cells from oxidative damage. An antioxidant is defined as “any substance that delays, prevents or removes oxidative damage to a target molecule” [23,36]. The antioxidant network fighting against the damaging potential of high levels of oxidants consists of two classes: (i) antioxidant enzymes and (ii) low-molecular-mass antioxidants [20,22,23].

## 3. Biomimetic Chemistry of Oxidative DNA Damage: cPu vs. 8-oxo-Pu

DNA is vulnerable to the attack of HO^•^ radicals, causing chemical modifications of nucleobases or nucleotides, abasic sites, and DNA strand breaks, which have been identified in living organisms [6]. These modifications are similar to those observed in vitro. Biomimetic experiments can be instrumental in understanding such processes’ dynamics and competitiveness issues [6]. The rate constant for the reactions of HO^•^ radicals with DNA has been reported to be ca. 2.5 × 10^8^ M^−1^ s^−1^ [50]. Recently, a rate constant of 1.4 × 10^10^ M^−1^ s^−1^ was determined for the reaction of HO^•^ with the double-stranded oligonucleotide (ds-ODN) containing GC pairs, i.e., the palindromic 5′-d(GCGCGC)-3′), and a value of 2.3 × 10^9^ M^−1^ s^−1^ can be estimated for the reaction of HO^•^ with the G:C pair, which is ~5-fold smaller than the sum of the two single nucleoside rate constants participating in the pair [51]. The site of attack is both the H-atom abstraction from the 2′-deoxyribose units and the addition to the base moieties, the latter accounting for ~85% of attacks in naked DNA [52]. The order of reactivity of HO^•^ radicals towards the various H-atoms of the 2′-deoxyribose moiety in DNA is widely accepted to be H5′ > H4′ > H3′ ≈ H2′ ≈ H1′, which goes along with the exposure of H-atoms to the solvent [53,54]. It is estimated that ca. 7% of HO^•^ radicals abstract the H5′ in naked DNA [55]. The fate of the resulting C5′ radical depends on its surrounding environment. Several studies have focused on the selective generation of C5′ radicals, yielding valuable quantitative data [56].

**Figure 2:**
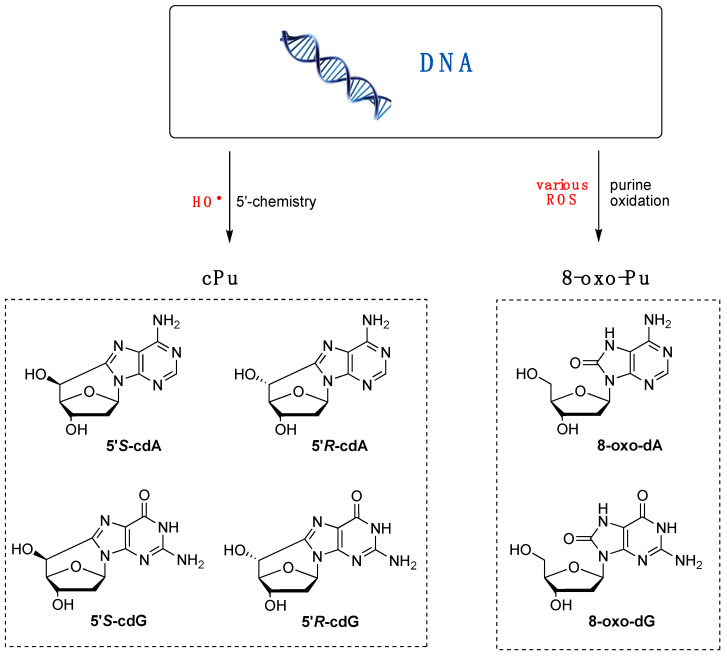
Purine lesions are formed after oxidatively induced DNA damage in vivo and in vitro; (left part) structures of 5′,8-cyclo-2′-deoxyguanosine (cdG) and 5′,8-cyclo-2′-deoxyadenosine (cdA) in their 5′*S* and 5′*R* diastereomeric forms generated by H5′ abstraction from HO^•^ radical; (right part) structures of 8-oxo-7,8-dihydro-2′-deoxyguanosine (8-oxo-dG) and 8-oxo-7,8-dihydro-2′-deoxyadenosine (8-oxo-dA) generated by ROS-mediated oxidation, also including the HO^•^ radical.

Various purine (Pu) lesions were observed in vitro due to oxidatively generated DNA damage [1,2]. Among these are the two 8-oxo-Pu lesions (8-oxo-dA and 8-oxo-dG) deriving from the oxidation of the base and the four cPu lesions (cdA and cdG in their 5′*R* and 5′*S* diastereoisomeric forms) originating from the C5′ radical chemistry of 2′-deoxyribose unit (Figure 2) [57,58]. The relative yields of these lesions depend on the reaction context, as evidenced by several comparative studies with various oxidants. For example, in isolated or commercial DNA, the 8-oxo-Pu and cPu lesions are detected in biomimetic experiments by specific generation of HO^•^ radicals, whereas 8-oxo-Pu is the product of other non-radical ROS.

**Figure 3:**
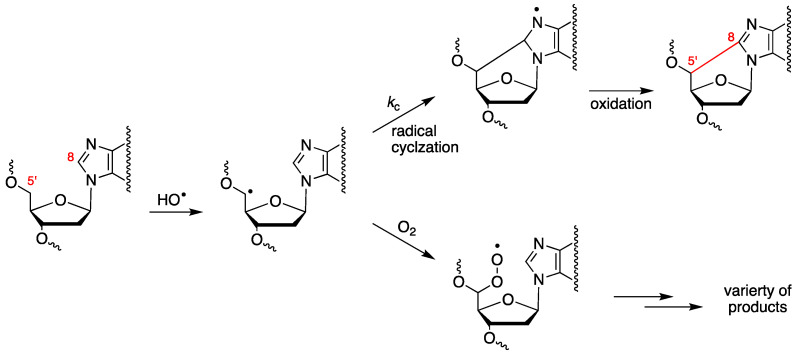
Purine 2′-deoxynucleotide moiety reacts with the HO^•^ radical, yielding the C5′ radical, whose fate is oxygen-dependent. Cyclization of the C5′ radical followed by oxidation leads to cPu lesions, whereas the reaction of the C5′ radical with oxygen provides the peroxyl radical that undergoes further transformations, including strand scission.

The abstraction of H5′ of purine moieties of DNA by HO^•^ radicals is estimated to be ca. 3% of all possible DNA attack sites [52]. The fate of the resulting C5′ radical is oxygen-dependent (see Figure 3). Under anaerobic conditions, the C5′ radical attacks the adenine or guanine moiety at the C8 position, forming a new covalent bond C5′–C8, followed by oxidation that leads to 5′,8-cyclopurines (cPu) [28,58]. By increasing the oxygen concentration, the cPu lesions decrease and the major pathway for DNA damage results in direct strand scission via the corresponding peroxyl radical [59,60,61].

Are ROS other than the HO^•^ radical able to generate C5′ radicals in DNA? O_2_^•−^ is unreactive in H-atom abstraction, the bond dissociation enthalpy (BDE) of C5′–H being 91.3 kcal/mol and that of ^−^O_2_–H being ca. 60 kcal/mol. Its conjugate acid, HOO^•^ (p*K*_a_ ~4.8), has a H-atom-abstracting ability comparable to that of the alkylperoxyl radicals (ROO^•^), i.e., 88–89 kcal/mol; however, it has been demonstrated that ROO^•^ cannot induce DNA strand scission [62,63]. Furthermore, HOO^•^ is present at only ca. 0.2% of the O_2_^•−^ concentration at pH 7.4.

HO^•^ radicals react with guanine and adenine moieties, leading to a variety of products that include the 8-oxo-Pu family (Figure 2) [28]. Other ROS transients, such as H_2_O_2_, ONOO^−^, singlet oxygen, or ROO^•^ radicals, can generate 8-oxo-Pu lesions [64,65]. The 8-oxo-dG is a major DNA lesion caused by oxidative stress, and it is further oxidized to other deleterious secondary DNA lesions [28]. 8-oxo-7,8-dihydro-2′-deoxyguanosine (8-oxo-dG) and 8-hydroxy-2′-deoxyguanosine (8-HO-dG) are the same molecule in two different tautomeric forms. Both acronyms, 8-oxo-dG and 8-HO-dG, are used in the literature, although NMR studies on a 12-mer ds-ODN indicated that 8-oxo-dG is the exclusive species with respect to its tautomer [66]. In this review, the acronyms 8-oxo-dG and 8-oxo-dA are used.

**Figure 4:**
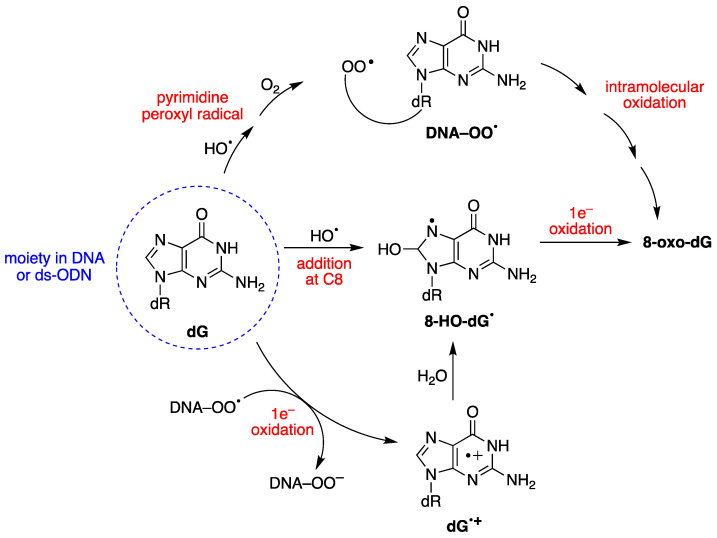
Mechanism for the oxidatively generated 8-oxo-dG in DNA or ds-ODNs; three pathways contribute to forming 8-oxo-dG, the two major ones being oxygen-dependent.

8-oxo-dG formation results from the initial attack by HO^•^ radicals or through a one-electron oxidation step [28]. The mechanism of 8-oxo-dG formation by the reaction of HO^•^ with ct-DNA [67,68], as well as with 21-mer ds-ODN [69,70], has been investigated in detail, in the absence or presence of different oxygen concentrations. It was demonstrated that three pathways contribute to forming 8-oxo-dG moiety, the two major ones being oxygen-dependent and a minor one being oxygen-independent, as shown in Figure 4 [67,68,70]. The minor path (5–10%) is the direct addition of HO^•^ to the C8 position of the guanine moiety to form 8-HO-dG^•^, followed by one-electron oxidation. The HO^•^ attacks are mostly non-selective (90–95%), generating diverse carbon-centered radicals that, in the presence of O_2_, are converted into corresponding peroxyl radicals, DNA–OO^•^ [52]. DNA–OO^•^ radicals produce two types of reactions in approximately equal amounts: (i) an intramolecular oxidation through addition of the peroxyl radical onto the C8 of a vicinal guanine base (see Figure 4) [67,68]; (ii) an intramolecular electron transfer leading to DNA^•+^, followed by hole transfer to [G:C]^•+^ pair, hydrolysis to give the 8-HO-dG^•^, and further oxidization to 8-oxo-dG [71]. Model studies on ODNs have reported the transient generation of pyrimidine peroxyl radicals, which add to the C8 position of a vicinal guanine base [72,73]. In these experiments, the formation of 8-oxo-dA was several times lower than that of 8-oxo-dG [67,69]. This lower yield of 8-oxo-dA formation was attributed to the absence of a one-electron oxidation pathway, and to a lesser extent, to the involvement of an intramolecular DNA–OO^•^ radical on the adenine base.

**Figure 5:**
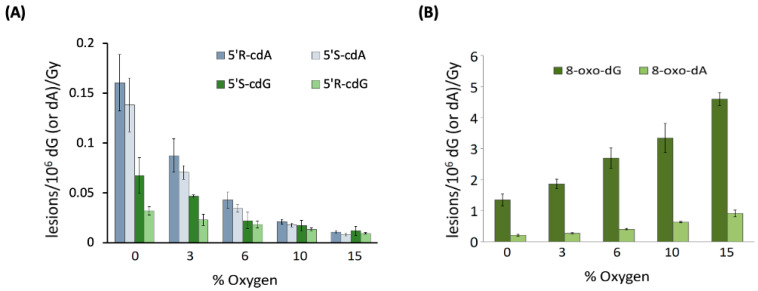
Oxygen-dependent radiation induced formation of purine lesions in ds-(ODN1/ODN2), where ODN1: 5′-GGG TTA GGG TTA GGG TTA GGG-3′ and ODN2: 5′-CCC TAA CCC TAA CCC TAA CCC-3′; cPu (**A**) and 8-oxo-Pu (**B**) lesions per 10^6^ dG (or 10^6^ dA) per Gy of γ-irradiation (in the presence of N_2_O) in the range of 0 to 60 Gy. Adapted from ref. [70] with permission of the American Chemical Society.

When examining 21-mer ds-ODNs [70], it was observed that increasing oxygen concentrations significantly reduced the levels of cdA and cdG. Figure 5A illustrates the variations in the levels of each of the four distinct cPu lesions per 10^6^ nucleosides (either dA or dG) per Gy of irradiation as oxygen concentration increases. This finding supports the notion that the C5′ radical has competing pathways—either cyclization or addition to molecular oxygen (see Figure 3). Rate constants for the cyclization steps were found to be in the range of 0.8–4.9 × 10^4^ s^−1^ through competition between the C5′ radical cyclization and the oxygen trapping. Moreover, it is noteworthy that the oxygen concentration did not influence the 5′*R*/5′*S* ratio for both cdG and cdA. As illustrated in Figure 5B, the reaction of HO^•^ radicals with 21-mer ds-ODNs results in an increased production of both 8-oxo-dG and 8-oxo-dA as the concentration of O_2_ rises. The levels of 8-oxo-dG were found to be six times higher than those of 8-oxo-dA. It is worth highlighting that increasing O_2_ concentrations led to reduced amounts of the four cPu lesions (Figure 5A) and excess amounts of the two 8-oxo-Pu lesions (Figure 5B). The 8-oxo-Pu lesions were only four times higher than the cPu ones in the absence of O_2_ but became 130 times more than cPu lesions at 15% O_2_, which corresponds to an O_2_ concentration of 2 × 10^−4^ M [70].

Comparison of the HO^•^ radical reactivity towards mtDNA and tDNA in an “isolated” context was addressed by the simultaneous measurement of 8-oxo-Pu and cPu lesions. Total (t) DNA is equal to nuclear (n) DNA plus mitochondrial (mt) DNA. The amount of 8–oxo–Pu and cPu lesions for the model reactivity of HO^•^ radical towards mtDNA and tDNA, isolated from wild-type CS1AN–wtCSB cells, under deoxygenated conditions was found to be (units of lesions/10^7^ nucleosides/Gy) 40.3 for 8–oxo–Pu and 19.0 for cPu in tDNA, and 4.6 for 8–oxo–Pu and 2.5 for cPu in mtDNA [74]. The 8–oxo–Pu was nearly twice the cPu values in both genomic materials, whereas 8–oxo–Pu and cPu were 8.8 and 7.6 times higher in tDNA than in mtDNA. These results under biomimetic conditions contribute to a better understanding of HO^•^ radical reactivity in distinct genetic pools, such as mtDNA and nDNA, highlighting the effects of different helical topology. In this respect, purine oxidation by the HO^•^ radical is highly dependent on tertiary ds-DNA helical forms having a supercoiled, open circular, and linear conformation, and that greater damage occurs towards the extended B-DNA topology for both 8-oxo-Pu and cPu [75].

## 4. The Protocol for the Simultaneous Quantification of Six Purine Lesions in DNA

Experimental protocols quantifying DNA lesions are numerous, the mass-spectrometry-based platforms being the most representative [65,76,77]. The use of genetic material, going down to the single nucleoside level by hydrolysis, and liquid chromatography–tandem mass spectrometry (LC–MS/MS), using isotopically labeled reference compounds [78,79], confer the advantage of specificity and repeatability, providing lower limits of detection (LOD) and quantification (LOQ) as well as precise measurement of DNA lesions [80]. Major efforts were devoted to the simultaneous quantification of purine lesions (four cPu and two 8-oxo-Pu) in cellular DNA, with the choice of the most appropriate assays, including suitable conditions of DNA extraction, hydrolysis, and base quantification [81,82,83]. Initially, we developed a protocol for the four cPu lesions [81,82] and later included the two 8-oxo-Pu lesions [83].

**Figure 6:**
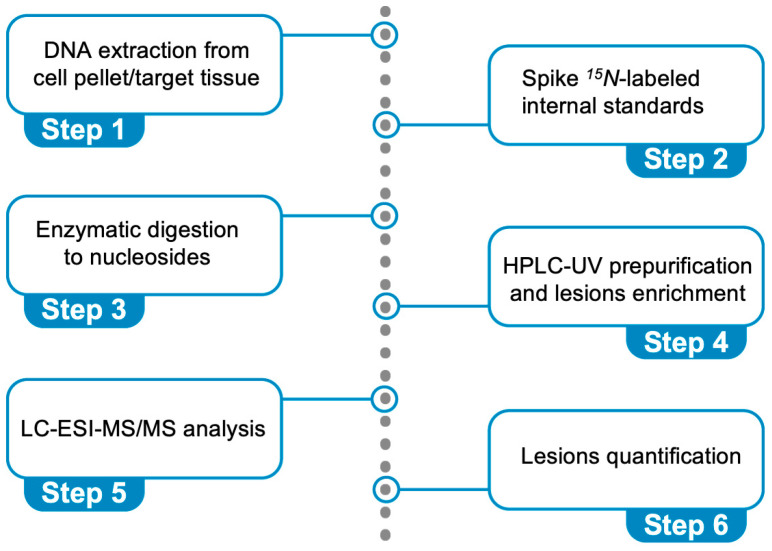
Protocol for the simultaneous quantification of the six oxidative purine lesions (see Figure 2) in DNA using LC-MS/MS analysis.

Figure 6 describes the steps: (i) DNA extraction from a cell pellet or a target tissue (addition of chelating agents and antioxidants), (ii) spiking ^15^*N*-labeled internal standards (addition of deaminase inhibitors and antioxidants), (iii) enzymatic digestion of DNA to nucleosides, (iv) HPLC-UV pre-purification and lesion enrichment (fraction collection), (v) quantification through liquid chromatography–tandem mass spectrometry (LC-MS/MS) analysis, and (vi) lesion quantification. It is worth underlining that the cPu lesions are stable compared with other oxidatively generated DNA adducts and cannot be artificially produced during DNA isolation. We optimized this protocol using model experiments of HO^•^ radical reactions with ds-ODNs or commercially available ct-DNA [69,70]. The data reported in Figure 5A,B refer to cPu and 8-oxo-Pu, respectively, for the reaction of HO^•^ radicals (generated by γ-radiolysis) with the 21-mer ds-(ODN1/ODN2), using our analytical protocol [70]. The reaction of HO^•^ radicals (generated by γ-radiolysis) with ct-DNA in aqueous solutions in the absence of O_2_ shows the level of total 8-oxo-Pu (8-oxo-dG/8-oxo-dA = 7.7) to be 40-fold in excess of total cPu lesions (5′*R*-cdG/5′*S*-cdG = 4.5 and 5′*R*-cdA/5′*S*-cdA = 1.2) [69]. In a recent article [83], we described the protocol in detail, including limitations and troubleshooting.

To determine the levels of six specific DNA lesions, we used this protocol on genomic and/or mitochondrial DNA extracted from cell cultures of various neurodegenerative disorders (further discussed in Section 6). Additionally, we applied it to DNA from estrogen-receptor-alpha-positive (ER-α) MCF-7 and triple-negative MDA-MB-231 breast cancer cell lines [84]. We also compared lesion levels in tissues (liver, kidney, and brain) of immunodeficient (SCID) xenografted mice with and without tumor implantation at different ages (4 and 17 weeks) [85]. Furthermore, we analyzed genetic material from tissue biopsies of patients with inflammatory bowel diseases (IBDs) and severely obese individuals in comparison to control samples [86].

## 5. Repair and Mutagenicity Studies: The Role of Nucleotide Excision Repair (NER)

Cells have developed a complex DNA repair network to manage oxidative insults and restore genome integrity [3,4,5]. DNA repair defects are linked with a wide spectrum of diseases, including cancer, premature aging, and neurological disorders [87]. BER and NER are cellular pathways for recognizing and repairing oxidatively induced DNA lesions [3,87]. BER is the primary system that processes base damage by creating an abasic site, e.g., the 8-oxo-dG lesion [88,89]. NER machinery is a widespread mechanism for repairing various helix-distorting DNA lesions, such as bulky DNA adducts caused by ultraviolet radiation and intra-strand crosslinks [90,91,92]. Evidence has also been provided on removing some oxidation-induced purine DNA lesions involving the overlap between these pathways [93]. NER is split into two sub-pathways: transcription-coupled (TC-NER), which repairs transcriptionally active domains, and global genome (GG-NER), which removes lesions from the whole genome [94,95]. Recent research using patient cells with specific NER gene defects and newly created knockout models has helped define the roles of GG-NER and TC-NER in repairing synthetic cdA and cdG lesions [96]. Defects in these sub-pathways may lead to clinical conditions such as xeroderma pigmentosum (XP) and Cockayne syndrome (CS). The NER pathway comprises many proteins (including the XP proteins (A through G) and the CSB proteins).

Biochemical studies demonstrated that the cPu lesions are not BER substrates but fairly good substrates of NER [97,98,99,100,101]. The cPu lesions cause structural deformation [102], and their transcription-blocking potential towards human RNA polymerase II have been reported [103]. The relative NER incision efficiencies of the four cPu lesions were measured in a 147-mer ds-ODN under the same sequence context in human HeLa cell extracts for a direct comparison [104]. The cdA and cdG lesions were excised with similar efficiencies by NER, the 5′*R* diastereomeric lesions being about twice as efficient as the 5′*S* diastereomeric lesions. Molecular modeling and dynamics simulations show that the cPu lesions cause more significant local distortion of the DNA backbone and a greater disruption of local van der Waals stacking interactions in 5′*R* than in 5′*S* diastereomers. The locally impaired stacking interaction energies correlate with relative NER incision efficiencies. Overall, NER is 2–4 times less effective at repairing cPu lesions than other bulky DNA adducts [104]. The relative NER efficiencies towards the cPu at the level of nucleosomes have also been addressed; the complete NER resistance of the cPu lesions in human HeLa cell extracts is attributed to the size of the cPu, which is similar to natural nucleotides [105]. Such a resistance means an accumulation of these lesions in the genome, which leads to the inhibition of DNA and RNA polymerase activities, DNA binding of transcription factors, replication fork stalling, and transcription termination [97,106,107]. Cells have evolved mechanisms to bypass these lesions through DNA repair, translesion DNA polymerases, and RNA polymerase II to address the challenges posed by cPu lesions. The DNA repair polymerases (pol) that can bypass cdA lesions include pol β, pol η, pol ι, and pol ζ [108,109,110,111,112,113]. Nonetheless, bypassing cPu lesions by DNA and RNA polymerases can also incorporate wrong nucleotides or result in the stalling of DNA and RNA synthesis [108,110,111,113,114,115]. For example, pol β can get stuck after inserting a dT opposite cdA, generating single-strand breaks [110]. On the other hand, RNA polymerase II and pol β can also cause multi-nucleotide deletion and repeat deletion to bypass cdA lesions during transcription and BER [109,111]. Recent findings show that human repair DNA polymerases pol β and pol η can incorporate the 5′*R*-cdA and 5′*S*-cdA lesions and create a cdA:dC mismatch [116]. Furthermore, the incorporation of these two lesions can be fully extended by pol β and ligated by LIG I, suggesting that cPu lesions can be embedded in the human genome from the damaged nucleotide pools [116].

In summary, the accumulation of cPu lesions in the human genome can lead to mutations and genome instability, which are linked to various diseases and conditions, including aging, inflammation, cancer development, and neurodegenerative disorders [57,117,118,119].

## 6. Oxidative Stress Causes Purine DNA Damage in Biological Models of Neurodegenerative Disorders

### 6.1. Xeroderma Pigmentosum (XP)

Xeroderma pigmentosum (XP-complementation group A–G) is an autosomal recessive genetic disorder associated with NER deficiency. As indicated in the previous section, NER is fundamental in repairing various helix-distorting or bulky DNA damage [91,92]. For example, the sunlight-induced photoproducts remain unrepaired, increasing the incidence of skin cancers [120,121,122]. The most severe cases of XP neurological disease are seen in XP-A patients, where defects emerge between the ages of 2 and 8. These cases present microcephaly, mild cognitive loss, followed by cerebellar variations and, ultimately, neuropathy [123]. XP-A is one of the 30 proteins involved in GG-NER and TC-NER; XP-A plays a central role in both sub-pathways, which cause different disease phenotypes [7,124,125]. However, many XP patients also display early-onset neurodegeneration, which leads to premature death. The mechanism of neurodegeneration is unknown.

Since exogenous ultraviolet radiation cannot reach the human brain, endogenous DNA lesions like the cPu, which are repaired exclusively by NER, have been assumed to elicit XP-related neurodegeneration [97,98,126]. cPu lesions either completely block RNAPII, causing transcription inactivation and neuronal death, or partially lead to mutant RNA transcripts and a deficient translation process [117,127]. Regarding the 8-oxo-Pu, in particular 8-oxo-dG lesions, earlier studies have shown that XPA-silenced cells, compared to the wild type (wt), are characterized by enhanced steady-state ROS levels and accumulation of 8-oxo-dG in their genome [128,129].

The enzyme-linked immunosorbent assay (ELISA) has been extensively employed for different biological applications, including identifying DNA adducts. However, the ELISA approaches regarding 8-oxo-dG overestimate the levels and therefore are inappropriate for an accurate quantification [130]. Using an XP group-A gene-knockout (*Xpa*^−/−^) mouse model, an ELISA test for the quantification of cdA (5′*R*-cdA and 5′*S*-cdA together) in DNA was generated by a monoclonal antibody (CdA-1) [131,132]. Initially, the detection level of the ELISA was as low as 10 lesions/10^6^ Nu in a 0.5 μg DNA sample, which was inadequate to evaluate different levels of cdA in tissues between *Xpa*^−/−^ and control wt mice [131]. Further optimization increased the ELISA sensitivity to a detection limit of 1 cdA lesion/10^6^ Nu [132]. Indeed, the improved ELISA showed that cdA lesions accumulate with age in the liver, kidney, and brain tissues of *Xpa*^−/−^ and of wt mice, but there were significantly more cdA lesions in *Xpa*^−/−^ mice than in wt mice at 6, 24, and 29 months of age [132]. These findings suggest that the brain’s age-dependent accumulation of endogenous cPu lesions may be critical for XP neurological abnormalities. However, a significant disadvantage of ELISA is the absence of structural information and potential lesion overestimation compared with mass spectrometry approaches.

**Figure 7:**
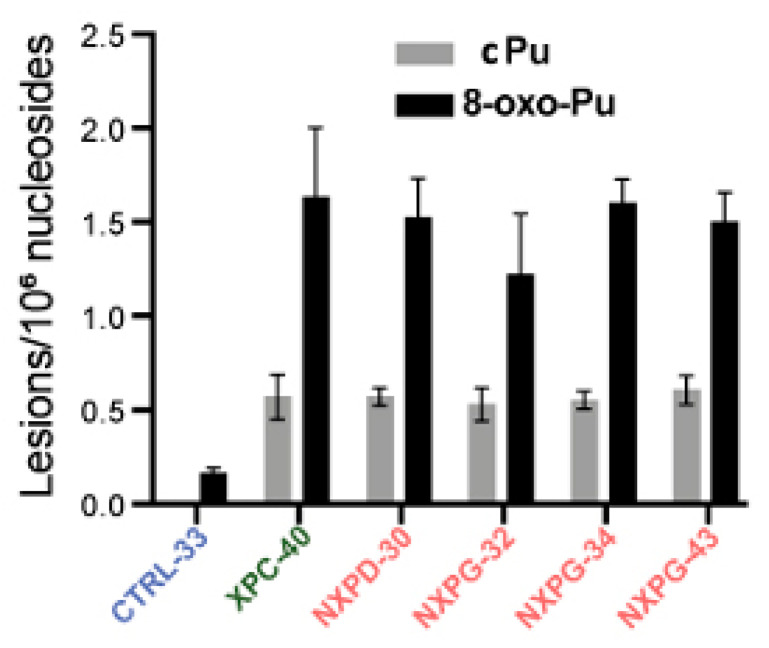
Comparison between cPu and 8-oxo-Pu levels (lesions/10^6^ nucleosides) in XP samples with neurodegeneration (red; NXP with *XPD* or *XPG* mutant), XP without neurodegeneration (green; XPC-40) and healthy controls (blue; CTRL-33). Adapted from ref. [133] with permission from Elsevier.

A recent investigation reported on XP neurodegeneration using human-induced pluripotent stem cells (hiPSC) derived from multiple XP complementation patients and healthy relatives, performing functional multi-omics on samples during neuronal differentiation [133]. Evidence is given of endoplasmic reticulum stress and marked oxidative DNA damage in XP neurons. The hiPSCs were obtained from one healthy control and four XP patients (groups D and G) with different cerebral disabilities but no signs of accelerated aging. After differentiation into neurons in triplicate, DNA was isolated and analyzed for the cPu and 8-oxo-Pu lesions, using the protocol mentioned in Section 4, on neurons from XP patients with or without neurodegeneration and healthy controls [133]. Figure 7 illustrates the substantial accumulation of oxidized nucleosides cPu and 8-oxo-Pu on several XP cell lines (XPC-40, NXPD-30, NXPG-32, NXPG-43, and NXPD-93). Compared with controls (CTRL-33), substantially elevated cPu lesions were observed in XP neurons with (NXPD or NXPG) or without (XPC-40) neurodegeneration. Increased 8-oxo-Pu in XP neurons was also found, the ratio 8-oxo-dG/8-oxo-dA being ~5. However, the fold increase was more significant for cPu than 8-oxo-Pu, resulting in an 8-oxo-Pu/cPu ratio of 2–3. For comparison, in the biomimetic chemistry of HO^•^ radicals with ct-DNA, the level of 8-oxo-Pu was found to be ~40-fold in excess of cPu in the absence of O_2_ [69], and in ds-ODN, the 8-oxo-Pu was 130 times more than cPu at 15% O_2_ concentration (see Figure 5) [70]. The 8-oxo-Pu lesions observed in controls agree with prior reports that hiPSCs are exposed to oxidative damage [134,135].

**Figure 8:**
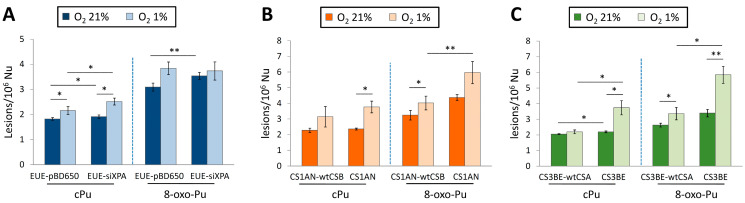
Comparison between cPu and 8-oxo-Pu levels (lesions/10^6^ nucleosides) in DNA extracted from various cells in aerobic (21% O_2_) and hypoxic (1% O_2_) conditions; (**A**) EUE-pBD650 (wt) and EUEsiXPA (*XPA* gene silenced by 80%) cells; (**B**) CS1AN-wtCSB and CS1AN (deficient) cells; (**C**) CS3BE-wtCSA and CS3BE (deficient) cells. * Denotes a statistically significant difference (*p* < 0.05) between the groups, ** denotes a statistically significant difference (*p* < 0.01) between the groups. Data from references [136,144].

The cPu and 8-oxo-Pu were simultaneously quantified in wt (EUE-pBD650) human embryonic epithelial cell lines and the same line, where the XPA gene was silenced by 80% (EUE-siXPA), under different oxygen tensions [136]. Table 1 reports the mean values (lesions/10^6^ Nu) of the six purine lesions under aerobic (21% of O_2_) and hypoxic (1% of O_2_) conditions. Figure 8A compares the sum of four cPu and the sum of two 8-oxo-Pu lesions. The 8-oxo-Pu levels were only ~1.7-fold higher than the cPu in both cell types, and both wt and silenced cell lines accumulated high levels of lesions under hypoxic conditions. Interestingly, the iron (Fe) levels were significantly higher in XPA-defective compared to wild-type cells (see Section 1 for the role of Fe^2+^ in the chemistry of ROS).

The results presented above on XP indicate that cPu lesions contribute to DNA damage in NER-deficient cells. This unrepaired oxidative DNA damage accumulates over time in terminally differentiated post-mitotic cells such as neurons and has deleterious effects on transcription and apoptosis regulation, resulting in neurodegeneration [137].

### 6.2. Cockayne Syndrome (CS)

Cockayne syndrome (CS) is a multi-system disorder associated in many patients with defects in NER [94]. CS provokes early aging and gradual neurological dysfunctions. Over 90% of CS cases are due to mutations in the *CSA* or *CSB* genes. A key aspect of CS cells is the defect in the TC-NER sub-pathway, which is responsible for removing lesions in the transcribed strand of actively transcribed genes [94]. Even though CSA and CSB patients present similar clinical characteristics, the proteins involved have distinct activities and functions in the TC-NER process. A key insight into the severe neurological abnormalities associated with CS was the discovery that the defect in TC-NER affects lesions repaired by NER and extends to oxidative DNA lesions, which are typically repaired by BER [95].

The sensitivity of CS cells to oxidatively generated DNA damage underscores the crucial role of CSA and CSB proteins in removing these lesions. Research has shown that the CSA protein in untreated human fibroblasts regulates the steady state level of 8-oxo-dG [138] and that keratinocytes from CSA patients accumulated 5′*S*-cdA under standard atmospheric oxygen tension [139]. Accumulation of 5′*S*-cdA in organs of CSB knockout mice has also been reported [140]. Furthermore, the CSB protein has been shown to enhance APE1 activity and to protect against agents that induce BER intermediates [141,142]. These findings highlight the importance of CS proteins in the recognition, signaling, and processing of single-strand breaks (SSBs) and double-strand breaks (DSBs), which are relevant lesions in neurodegenerative disorders [143].

Cells from CS patients, with mutations in *CSA* or *CSB* genes, present elevated levels of ROS and are defective in the repair by NER of various helix-distorting DNA lesions. The cPu and the 8-oxo-Pu lesions were ascertained in both wild-type (CS3BE–wtCSA) and (CS1AN–wtCSB) cell lines, and their defective counterparts CS3BE and CS1AN [144]. Like in XPA cells described above, the role of oxygen concentration in DNA damage was also reported for CSB and CSA cells. Table 1 reports the mean values (lesions/10^6^ Nu) of the six purine lesions under aerobic (21% of O_2_) and hypoxic (1% of O_2_) conditions. Figure 8B,C illustrate the sum of four cPu and two 8-oxo-Pu lesions for CSB and CSA, respectively. cPu levels were comparable to 8-oxo-Pu levels in all cases, i.e., within the range of 2–6 lesions/10^6^ Nu, and there was a substantial increase from aerobic to hypoxia. Comparing the accumulations, it can be seen that (i) the 8-oxo-Pu level is 1.3–1.9-fold higher than cPu for CS1AN-wtCSB and CS1AN cells (Figure 8B), and (ii) the 8-oxo-Pu level is 1.3–1.7-fold higher than cPu for CS3BE–wtCSA and CS3BE cells (Figure 8C). In hypoxic conditions, CSB- and CSA-defective cells showed higher levels of the four cPu lesions, compared to regular counterparts, and a significant increase in 8-oxo-Pu [144].

**Table 1:** The levels (lesions/10^6^ nucleosides) of four cPu and two 8-oxo-Pu lesions in DNA isolated from EUE-pBD650 (wt) and EUE-siXPA (*XPA* gene silenced by 80%), CS1AN-wtCSB and CS1AN (deficient), and CS3BE-wtCSA and CS3BE (deficient) cells in aerobic (21% O_2_) and hypoxic (1% O_2_) conditions *.

Cell Type		O_2_	5′*R*-cdG/5′*S*-cdG	5′*R*-cdA/5′*S*-cdA	8-oxo-dG/8-oxo-dA
XPA	EUE-pBD650	21%	0.3/0.9	0.5/0.2	2.6/0.5
		1%	0.3/1.0	0.6/0.2	3.2/0.7
	EUE-siXPA	21%	0.3/0.9	0.5/0.2	3.0/0.5
		1%	0.4/1.2	0.7/0.2	3.1/0.6
CSB	CS1AN-wtCSB	21%	0.5/0.9	0.6/0.2	2.7/0.6
		1%	0.7/1.3	0.8/0.3	3.3/0.7
	CS1AN	21%	0.5/0.9	0.7/0.2	3.8/0.6
		1%	0.9/1.5	1.0/0.4	4.8/1.1
CSA	CS3BE–wtCSA	21%	0.4/0.9	0.6/0.2	2.0/0.6
		1%	0.4/0.9	0.6/0.3	2.7/0.7
	CS3BE	21%	0.4/0.9	0.7/0.2	2.7/0.8
		1%	0.8/1.2	1.3/0.4	4.4/1.4

* Mean values of three sample measurements; for the standard deviation, see the original articles [136,144].

A similar trend of increased levels of cPu and 8-oxo-Pu lesions was observed in defective CSB (CS1AN) and CSA (CS3BE) compared to normal fibroblasts CS1AN-wtCSB and CS3BE-wtCSA (Figure 8B,C), as well as EUE-siXPA (XPA gene was silenced by 80%) compared to wt human embryonic epithelial cell lines EUE-pBD650 (Figure 8A) under aerobic conditions (O_2_ 21%). It is noticeable that the relatively small differences in the levels of cPu vs. 8-oxo-Pu and the increased levels of cPu and 8-oxo-Pu lesions go from aerobic (21% O_2_) and hypoxic (1% O_2_) conditions (Figure 8). The same trends are observed for the six individual lesions (Table 1). Several studies support the role of oxidative DNA damage in neurodegenerative diseases and aging processes, and the aim was to elucidate the role of oxygen incubation conditions when cellular repair capacity is affected. It is worth mentioning that there is a link between hypoxia and neurodegeneration, but the mechanisms by which this occurs are not yet completely understood [9,145]. Hypoxia has been reported to increase ROS generation and induce neuroinflammation [145,146]. Moreover, hypoxia (1% O_2_) is known to cause a rapid increase in the expression of several genes and, subsequently, induce increased levels of intracellular ROS and DNA damage accumulation. The accumulation of cPu lesions observed under hypoxic conditions indicates that XP and CS proteins are involved in cPu repair through the NER pathway. cPu lesions are transcription-blocking, and the build-up of this type of oxidative DNA damage in actively transcribed genes has been linked with neuronal death [147]. It is well established that the neurological defects observed in CS patients differ significantly from those in XPA patients, characterized by extensive neurodegeneration [148]. Thus, the accumulation of cPu lesions plays a crucial role in the neurological symptoms of these syndromes. Moreover, the abundance of cPu lesions in CSB and CSA cells, like in XPA, endorses their role as candidate markers for pathologies with NER defects.

### 6.3. Mitochondria DNA (mtDNA) in Cockayne Syndrome (CS)

mtDNA is a circular molecule of approximately 16.5 kb in length. It exists in a compacted DNA–protein complex known as the mitochondrial nucleoid, which may provide some protection against ROS produced by the mitochondrial respiratory chain [149,150]. Similar to nuclear DNA (nDNA), mtDNA is highly susceptible to ROS, leading to accumulation of DNA modifications [151,152,153]. Increased oxidative damage in mtDNA has been linked to neurological degeneration, inflammasomes, tumor development, and cancer progression [154,155]. Among the various mtDNA repair pathways, the BER pathway has been well studied and shown to effectively remove certain types of oxidative DNA damage in the mitochondria, comparable to its efficiency in the nuclei [152]. Implications of other repair pathways remain unclear, although it is well established that NER does not occur in mitochondria [147]. Despite the constant exposure to ROS and the less protective pathways available in mitochondria, it remains unclear how the integrity of genetic information in this compartment is preserved [151,152,153]. However, mitochondria have evolved a unique mechanism which maintains mtDNA integrity through degradation of excessively damaged genomes followed by replication of intact/repaired mtDNA. This mechanism is not present in the nucleus and is enabled by multiple copies of mtDNA present in mitochondria. In mitochondria, a unique pathway occurs that is enabled by high redundancy of the mitochondrial DNA and allows for the disposal of damaged DNA molecules operating in this organelle [156].

**Figure 9:**
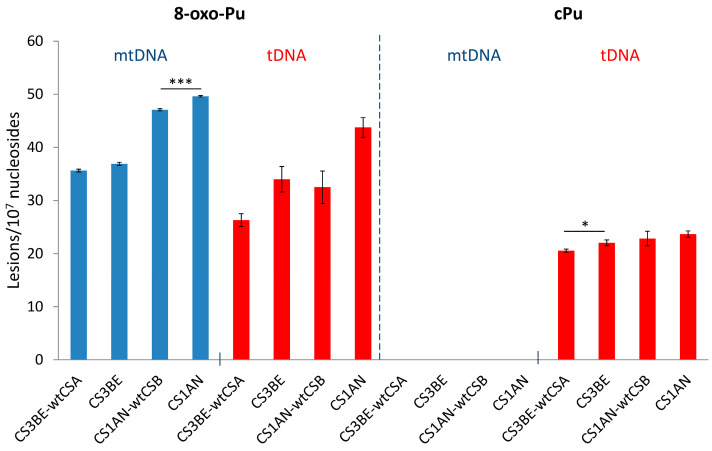
Comparison between 8-oxo-Pu and cPu levels (lesions/10^7^ Nu) in mtDNA (blue) and tDNA (red) samples extracted from CS1AN-wtCSB, CS1AN, CS3BE-wtCSA, and CS3BE cells. * Denotes a statistically significant difference (*p* < 0.05) between the groups, *** denotes a statistically significant difference (*p* < 0.001) between the groups. Taken from ref. [74].

The cPu and 8–oxo–Pu lesions were identified in the mtDNA of wtCSA and wtCSB cells, along with their defective counterparts CS3BE and CS1AN, in comparison to the corresponding total (t) DNA (t = n + mt). Figure 9 (left side) shows that the 8–oxo–Pu levels are comparable in both mtDNA and tDNA in defective cells and the wt counterparts of CSA and CSB cells. Figure 9 (right side) shows the presence of cPu lesions in the tDNA and their absence in mtDNA (at least a 100 times lower level than in tDNA), indicating an absence of HO^•^ radical reactivity within mtDNA [74]. The nonexistence of cPu lesions in the mtDNA of CS cells may involve specific mechanisms devoted to maintaining the mitochondrial genome integrity in view of the absence of NER in the mitochondria [155]. In the biomimetic model examining HO^•^ radicals with purified tDNA and mtDNA, it was found that the levels of all six purine lesions in tDNA were approximately eight times higher than those in mtDNA. This disparity suggests that HO^•^ radicals have different accessibility to mtDNA compared to nDNA, which can be attributed to variations in their helical topologies (cf. Section 3) [75]. In this respect, purine tDNA lesion levels in defective CSA cells overexpressing Parkin, a mechanism devoted to the removal of dysfunctional mitochondria [157,158], have an effect on 8-oxo-Pu rather than cPu [143], indicating that mitochondria do not play a role in the accumulation of cPu lesions.

### 6.4. 5′R and 5′S Diastereomers of cPu Lesions and the 8-oxo-dG/8-oxo-dA Ratio

The 8-oxo-dG and 8-oxo-dA measured across biological models of XP and CS cell lines indicate a higher accumulation of such lesions, going from aerobic to hypoxic conditions, with 8-oxo-dA increasing a little more than 8-oxo-dG (see Table 1). In all described cell lines, the 8-oxo-dG/8-oxo-dA ratio varies from 3.4 to 6.3, the majority being close to 5, which indicate a substantial amount of 8-oxo-dA. Although the 8-oxo-G is the most abundant and comprehensively studied DNA lesion resulting from oxidative damage, the analogous information of 8-oxo-dA on the cellular level is much less available. The accumulation of 8-oxo-dG, along with 8-oxo-dA, represents a significant threat to genomic stability [159].

The diastereomeric ratio (5′*R*/5′*S*) of cdG and cdA is a fascinating subject of mechanistic investigation on the occurrence of this free radical damage. The information reported in Table 2 for XPA, CSB, and CSA cells can be obtained from Table 1. In all cases, independently of the cell line type (wt or defective) and oxygen concentration, in cdG the 5′*S* configuration is always more abundant than the 5′*R* one, whereas in cdA, the 5′*R* is always more abundant than 5′*S.* For comparison, in the case of in vivo damage to the brain of tumor-bearing SCID mice (17 weeks) and colon tissue from inflammatory bowel diseases (IBD) patients, determined by the same analytical performance and uncertainty of the measurements, levels of cPu and 8-oxo-Pu similar to the in vitro models were reported, but with the diastereoisomeric ratios equal or in favor of 5′S (Table 2). At least two factors play a role in determining the diastereomeric ratios of cdG and cdA: (a) the local conformations at the reactive sites prior to C5′ radical cyclization, which increases the prevalence of one type of diastereoisomer [70], and (b) the NER efficiency for the repair of the diastereoisomers [104].

The cdA and cdG lesions are removed with comparable efficiency by NER; however the formation of the 5′*R*-diastereoisomers of cdA and cdG in the DNA backbone causes more significant distortion, becoming better substrates of NER than the corresponding *S* ones [104]. The measured values of the 5′*R*/5′*S* ratio for cPu lesions in cells (XPA, CSB, CSA, and XP neurons) compared with tissues reflect connections and distinct pathways between neurodegenerative diseases and pathologies with deficiencies in the NER process.

## 7. Conclusions

Most DNA damage in induced by oxidative metabolism, emphasizing the role of nucleic acid oxidation in the etiology and treatment of various diseases and aging processes. The accumulation of cPu as specific markers of HO^•^ radical damage and comparison with 8-oxo-Pu arising by various ROS are discussed with the results from several neurological cell types. Considering that cPu and 8-oxoPu DNA lesions do not suffer from stability issues and artifacts of other oxidatively generated DNA lesions, this review describes how the simultaneous detection of cPu and 8-oxoPu can be determinant to depicting the scenario of DNA exposure, highlighting the conditions where nucleotide excision repair (NER) is defective. A significant connection has been described between defective enzymatic repair mechanisms for oxidative DNA damage and oxygen concentration in various XP- and CS-deficient cell lines conditions. These results support the hypothesis that defective repair of oxidative DNA damage, particularly cPu, is involved in the clinical outcomes of neurodegenerative disorders.

## Figures and Tables

**Table 2:** Diastereomers ratios of cPu lesions.

Diastereomeric Ratios	XPA Cells	CSB Cells	CSA Cells	Mouse Tumor Brain ^1^	BDI Patients ^2^
5′*R*/5′*S* of cdG	0.3	0.5	0.5	0.7	0.16
5′*R*/5′*S* of cdA	2.9	3.0	3.0	1.0	0.4

^1^ From ref. [85]; ^2^ from ref. [86].

## Abbreviations

The following abbreviations are used in this manuscript:

BER	Base excision repair
CAT	Catalase
CS	Cockayne syndrome
cdA	5′,8-cyclo-2′-deoxyadenosine
cdG	5′,8-cyclo-2′-deoxyguanosine
cPu	5′,8-cyclopurine
ct-DNA	Calf thymus DNA
ds-ODN	Double-stranded oligonucleotide
ELISA	Enzyme-linked immunosorbent assay
GG-NER	Global genome nucleotide excision repair
GPx	Glutathione peroxidase
hiPSC	Human-induced pluripotent stem cells
MPO	Myeloperoxidase
mtDNA	Mitochondrial DNA
NER	Nucleotide excision repair
NOS	Nitric-oxide synthases
OS	Oxidative stress
8-oxo-Pu	8-oxopurine
Prx	Peroxiredoxin
Pu	Purine
ROS	Reactive oxygen species
SOD	Superoxide dismutase
TC-NER	Transcription-coupled nucleotide excision repair
wt	Wild type
XP	Xeroderma pigmentosum
